# *Amanita* Species Mushroom Poisonings — Northern California, November 2025–March 2026

**DOI:** 10.15585/mmwr.mm7520a2

**Published:** 2026-05-28

**Authors:** Kevin J. Brandecker, Chelsea Hayman, Kathy T. LeSaint, Rais Vohra, Asha Choudhury, M. Elizabeth Marder, Russell Bartlett, Tracy Barreau, Craig G. Smollin

**Affiliations:** ^1^Department of Emergency Medicine, University of California, San Francisco, California; ^2^California Poison Control System, San Francisco Division, San Francisco, California; ^3^California Poison Control System, Fresno/Madera Division at Valley Children’s Hospital, Madera, California; ^4^Department of Emergency Medicine, University of California San Francisco Fresno, Fresno, California; ^5^California Department of Public Health, Environmental Health Investigations Branch, Richmond, California ^6^Career Epidemiology Field Officer Program, CDC.

**Summary**

**What is already known about this topic?**

The resemblance of some toxic mushrooms to edible mushrooms can result in misidentification by persons who forage wild mushrooms. Eating amatoxin-containing mushrooms can result in liver failure and death.

**What is added by this report?**

During November 2025–March 2026, the California Poison Control System and California Department of Public Health responded to an outbreak of 39 cases of amatoxin mushroom poisoning in northern California, resulting in three liver transplantations and four deaths. Many cases occurred in persons who had previously foraged similar-appearing mushrooms in other countries. Collectively, the affected patients spoke at least six languages other than English.

**What are the implications for public health practice?**

Eating foraged mushrooms remains an activity with high risk for poisoning, especially during rainy seasons, when amatoxin-containing mushrooms can fruit widely. Educational materials in multiple languages might reduce harmful exposures.

## Abstract

The genus *Amanita* contains approximately 600 species of mushrooms, including some that produce amatoxins, which can lead to liver failure and death when ingested. Poisonous mushrooms often resemble and are difficult to distinguish visually from nonpoisonous, edible mushrooms. After above-average late November 2025 rainfall in California, regional mycologists observed numerous *Amanita* mushroom blooms in regional parks and area wildlands. On November 18, 2025, specialists at the San Francisco division of the California Poison Control System (CPCS) suspected amatoxin-containing mushroom poisoning in two patients who gastrointestinal symptoms and hepatotoxicity after eating foraged wild mushrooms. Over the next 17 weeks (November 18, 2025–March 17, 2026), 39 cases of suspected amatoxin mushroom poisoning in patients who had eaten foraged wild mushrooms were reported to CPCS. These 39 cases were characterized by a pattern of delayed onset of gastrointestinal symptoms and hepatotoxicity; 32 (82%) patients recovered, three (8%) required liver transplants, and four (10%) died. CPCS and the California Department of Public Health Toxicological Outbreak Program coordinated a response that included a statewide health advisory and educational materials translated into multiple languages for the public; collectively, the affected patients spoke at least six languages other than English. This is the largest reported outbreak of mushroom-associated hepatotoxic poisoning in California history and the largest in the United States in several decades. This was also the first outbreak of this size in which some persons ate *Amanita ocreata*, another poisonous *Amanita* species. The morbidity and potential lethality associated with amatoxin-containing mushroom ingestion is a serious public health concern. Educational materials, including for non–English-speaking communities, during the late fall to mid-spring when fruiting of amatoxin-containing mushrooms occurs, might reduce the number of poisonings.

## Investigation and Results

Although the *Amanita* genus comprises many edible mushrooms, the genus also includes some of the most toxic mushrooms worldwide. The *Amanita* mushrooms that are responsible for most deaths contain a group of highly potent hepatotoxins called amatoxins (*1*). Edible and toxic *Amanita* species can be difficult to distinguish based on appearance alone, especially for inexperienced foragers. The California Poison Control System (CPCS) typically receives fewer than five reported cases of suspected amatoxin mushroom poisoning each year (*2*); however, during December 2016, an outbreak involving 14 cases in northern California resulted in three liver transplantations, and during November 18–29, 2025, CPCS was notified of 11 suspected cases (*3*). CPCS recognized that an unusually large outbreak was occurring, initiated a case investigation, and organized a public health response in coordination with the California Department of Public Health (CDPH). This activity was reviewed by CDC, deemed not research, and conducted consistent with applicable federal law and CDC policy.* This report describes the findings from the investigation.

### Identification of Initial Cases

On November 16, 2025, a man aged 36 years (patient 1) and his sister, aged 38 years (patient 2) were evaluated in a local emergency department for abdominal pain, nausea, vomiting, and diarrhea (Table). Symptoms had begun 4 days earlier, several hours after they had eaten mushrooms foraged by another family member. Initial laboratory tests revealed elevated liver aminotransferase levels and a normal international normalized ratio. The treating clinicians contacted CPCS on November 18, who, in discussion with CPCS pharmacists and medica toxicologists, suspected amatoxin mushroom poisoning. Both patients recovered after inpatient treatment with intravenous (IV) fluid hydration and IV N-acetylcysteine and were discharged after 3 days. Regional mycologists reported very large blooms (superblooms) of *Amanita* species mushrooms in the San Francisco Bay Area after above-average rainfall in California during late November 2025. During November 21–24, three additional cases of suspected amatoxin mushroom poisoning were identified (patients 3, 4, and 5).

**TABLE:** Clinical and demographic characteristics of patients with *Amanita* species mushroom poisoning — northern California, November 2025–March 2026

Patient	Age, yrs (sex)	No. of mushrooms eaten	Symptom onset to ED visit, hrs	Initial AST/ALT,* units/L	Peak AST/ALT, units/L	Peak INR,^†^ units	Peak Cr,^§^ mg/dL	No. of days in hospital	Interventions received^¶^	Outcome
1	36 (M)	NA	96	873/2,135	1,512/2,135	1.3	0.9	3	NAC	Recovered
2	38 (F)	NA	96	147/117	147/117	NA	0.8	3	NAC	Recovered
3	56 (M)	1	29	67/81	215/274	1.1	1.4	3	NAC and vitamin C	Recovered
4	30 (M)	Multiple	24	80/102	720/594	1.2	1.0	4	Octreotide and biliary drainage	Recovered
5	43 (M)	2	10	64/70	4,474/6,269	4.8	1.7	8	Silibinin, NAC, AC, penicillin G, and octreotide	Recovered
6**	1.5 (F)	NA	NA	72/49	7,000/7,000^††^	5.5	0.2	11	Silibinin, NAC, AC, penicillin G, cyclosporine, octreotide, and biliary drainage	Recovered
7**	33 (M)	NA	NA	37/58	51/66	1.1	1.1	5	NAC, AC, penicillin G, and octreotide	Recovered
8**	37 (M)	4	NA	65/75	11,000/8,741	1.9	0.7	6	NAC, octreotide, and biliary drainage	Recovered
9**	43 (M)	NA	NA	132/59	4,906/1,804	1.4	0.4	5	Silibinin, NAC, penicillin G, vitamin C, cyclosporine, octreotide, and biliary drainage^§§^	Recovered
10**	43 (F)	NA	8	78/98	114/125	1.1	0.6	4	NAC, AC, penicillin G, and vitamin C	Recovered
11**	50 (M)	NA	11	50/49	3,146/5,250	8.7	1.4	16	Silibinin, NAC, AC, penicillin G, cyclosporine, and biliary drainage	Liver transplant
12	34 (M)	0.5	NA	707/498	7,599/8,575	2.0	2.1	NA	NAC and penicillin G	Recovered
13	25 (M)	NA	9	47/97	4,980/6,260	1.7	0.7	NA	Silibinin, NAC, penicillin G, and octreotide	Recovered
14	45 (M)	NA	9	1,069/587	6,173/1,406	4.6	2.8	3	NAC and penicillin G octreotide	Died
15	39 (F)	NA	9	24/33	564/532	1.1	0.6	5	Octreotide, NAC, and penicillin G	Recovered
16	31 (M)	NA	NA	265/281	4,936/5,168	2.2	1.3	9	Silibinin, NAC, penicillin G, and octreotide	Recovered
17	23 (F)	<1	63	310/791	310/791	1.3	0.8	3	NAC	Recovered
18	39 (M)	NA	NA	202/190	10,406/6,955	1.8	1.9	NA	NAC, penicillin G, octreotide, and biliary drainage	Liver transplant
19	35 (F)	NA	NA	66/61	10,000/10,000^††^	2.0	0.7	4	Octreotide and biliary drainage	Recovered
20	50 (M)	5–6	23	52/46	277/280	1.2	0.8	4	NAC and penicillin G	Recovered
21	54 (F)	“Handful”	20	32/23	744/971	1.1	0.6	5	NAC, AC, and penicillin G	Recovered
22	42 (M)	NA	NA	431/621	621/431	1.1	0.9	NA	NAC	Recovered
23	40 (F)	NA	16	23/23	866/1,066	1.0	0.6	NA	NAC, penicillin G, and octreotide	Recovered
24	54 (M)	NA	16	50/42	11,012/5,955	2.1	0.6	5	Silibinin, NAC, AC, penicillin G, octreotide, and biliary drainage	Recovered
25	41 (M)	NA	26	121/164	4,904/5,627	9.3	1.3	18	NAC and penicillin G	Liver transplant
26	33 (F)	NA	26	52/53	616/889	1.3	0.6	NA	Silibinin, NAC, AC, and penicillin G	Recovered
27	17 (F)	NA	26	98/106	4,251/6,495	7.0	0.7	8	Silibinin, NAC, and penicillin G	Recovered
28	31 (M)	NA	22	26/28	2,524/4,547	2.9	11.0	NA	NAC, penicillin G, octreotide, and biliary drainage	Recovered
29	59 (M)	NA	22	91/93	8,513/8,618	4.7	1.0	NA	Silibinin, NAC, penicillin G, and biliary drainage	Recovered
30	35 (M)	NA	44.5	9,800/7,800	9,869/8,771	1.5	NA	NA	NAC, penicillin G, and octreotide	Recovered
31	49 (M)	“Some”	15	90/133	5,283/5,743	5.6	1.5	6	NAC, AC, penicillin G, octreotide, and biliary drainage	Died
32	29 (M)	20	24	74/113	3,708/5,949	6.2	4.4	4	Silibinin, NAC, and penicillin G	Died
33	67 (M)	1	26	4,100/4,000	5,700/7,000^††^	6.8	4.2^¶¶^	12	NAC, penicillin G, and octreotide	Died
34	32 (M)	NA	12	33/39	214/396	1.3	1.3	4	NAC, penicillin G, and AC	Recovered
35	8 (M)	NA	17	205/238	205/238	2.4	0.5	4	NAC and AC	Recovered
36	6 (F)	NA	12	30/18	30/18	1.4	0.5	4	NAC and AC	Recovered
37	32 (F)	4	12	21/20	15,238/16,038	5.4	0.5	NA	Silibinin, NAC, AC, and penicillin G	Recovered
38	37 (F)	3	16	70/64	10,000^††^/2,200^††^	NA	0.6	NA	Silibinin, NAC, and penicillin G	Recovered
39	27 (NB)	2	62	1,530/3,861	1,530/3,861	1.4	1.1	44	NAC and penicillin G	Recovered

**Abbreviations:** AC = activated charcoal; ALT = alanine transaminase; AST = aspartate transaminase; Cr = creatinine; ED = emergency department; F = female; INR = international normalized ratio; M = male; NA = not available; NB = nonbinary; NAC = N-acetylcysteine.

* Normal AST = 15–41 units/L; normal ALT = 17–63 units/L.

^†^ Normal INR = 0.8–1.2 units.

^§^ Normal Cr = 0.8–1.2 mg/dL (adult); 0.3–0.5 mg/dL (child); and 0.5–0.8 mg/dL (adolescent).

^¶^ All patients received intravenous fluids and electrolytes. All treatments other than silibinin were used off-label and not approved for treatment of amatoxin poisoning in the United States.

** Part of a cluster of six patients in the same family.

^††^ Values exceed the maximum measurable level for that laboratory.

^§§^ Unsuccessful biliary drainage procedure.

^¶¶^ Patient with preexisting history of end-stage kidney disease.

### Additional Cases and Outcomes

During late November 2025–March 2026, CPCS received calls regarding an additional 34 cases of liver injury (patients 6–39). All cases occurred after patients ate foraged wild mushrooms.

**Fatal poisonings.** Four patients died after eating foraged wild mushrooms. Patient 14, a man aged 45 years, ate mushrooms foraged in a national park and died 4 days later. Two of his family members (patients 13 and 15) ate smaller quantities of the same mushrooms and had liver injury but recovered. Patient 31, a man aged 49 years, ate mushrooms similar to those he had eaten in Mexico and died after a 7-day hospital course. Patient 32, a man aged 29 years, ate 20 mushrooms he had foraged in a local forest. He was initially discharged from an emergency department with a diagnosis of gastroenteritis; however, he returned the next day and died 4 days later. Patient 33, a man aged 67 years, ate one large mushroom and died on hospital day 12 days.

***Amanita ocreata* poisonings**. Five cases of poisoning with a different species of amanita mushroom occurred in a family of four (a woman and a man, both aged 32 years, a boy aged 8 years, and a girl aged 6 years) (patients 34–37), and an unrelated person aged 28 years (patient 39). These persons experienced abdominal pain, nausea, vomiting, and diarrhea 8–12 hours after eating meals containing mushrooms foraged in two different regions of northern California. The family foraged mushrooms in a regional park, and the mushrooms eaten by patient 39 were foraged by another person in a national forest 150 miles (241 km) from where patients 34–37 foraged. In both instances, using photos provided by the patients, CDPH and regional mycologists identified the mushrooms as *A. ocreata*, also known as the western destroying angel (Figure).

**FIGURE:**
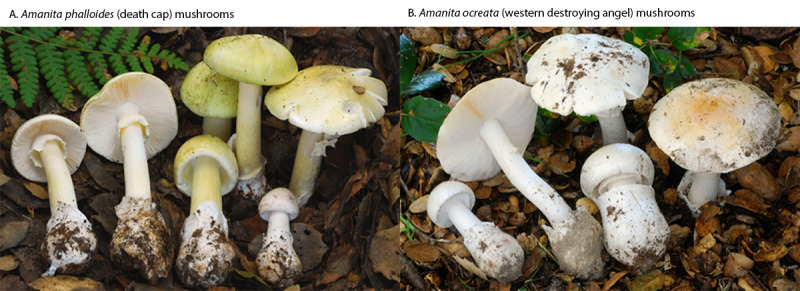
*Amanita phalloides* (A) and *Amanita ocreata* (B) mushrooms — northern California, November 2025–March 2026 Photos/California Department of Public Health

**Poisoning after eating found mushrooms.** Patient 38 was a woman aged 37 years who was experiencing homelessness and ate mushrooms she found in a bag left on top of a garbage can. She sought care at the hospital with gastrointestinal symptoms, developed mildly elevated aminotransferase levels, and later recovered. Because of the unusual history in this case, CDPH’s Laboratory Response Network for Chemical Threats, a national network coordinated by CDC, performed urine testing, which confirmed the presence of amatoxin.

## Public Health Response

After recognizing an outbreak of poisoning resulting from eating amatoxin-containing mushrooms, CPCS worked closely with the CDPH Toxicological Outbreak Program to coordinate a timely response. A case definition for wild mushroom poisoning was developed and included patients who 1) ate or likely ate wild mushrooms, 2) were hospitalized within 1 week of consumption, and 3) had evidence of liver injury consistent with amatoxin poisoning. On December 5, CDPH and CPCS released a statewide health advisory through the California Health Alert Network that included guidance to health care providers regarding recognition and treatment of patients with suspected amatoxin poisoning. The investigation revealed that multiple languages other than English were spoken by patients, including Spanish, Mixteco (an indigenous Mexican language), Mam (an indigenous Mayan language primarily spoken in Guatemala), Ukrainian, Russian, and Mandarin Chinese. Two patients were unhoused (patients 22 and 38), and one patient reported having eaten the mushrooms because of food insecurity. To support a public outreach campaign focusing on the dangers of foraging wild mushrooms, educational materials were developed and translated into the identified languages. Multilingual posters were disseminated through social media, and distributed to mycological societies, parks, and recreation contacts statewide. These posters advised against foraging for wild mushrooms, included warnings that eating wild mushrooms can result in liver failure and death, used clear graphics (including a globally recognized hazard symbol), highlighted the resemblance between poisonous and safe edible mushroom varieties, and explained how to seek medical help. The posters were displayed in areas where mushroom poisonings had occurred and other areas where *A. phalloides* or *A. ocreata* were observed to be growing. Both agencies jointly coordinated with local public health officers, hospital leaders, media outlets (including Spanish television and radio), mycological societies, and community-based organizations to disseminate public health messages and share updates about the outbreak and the response.

## Discussion

During November 2025–March 2026, CPCS was involved in the response to 39 cases of suspected amatoxin-containing mushroom intoxication across California, which resulted in three (8%) liver transplants and four (10%) deaths. This is the largest outbreak of mushroom-associated hepatotoxic poisoning ever reported in California and the largest in the United States in decades. Amatoxin-containing mushrooms account for >90% of mushroom poisoning deaths worldwide; even with medical intervention, amatoxin-containing mushroom poisoning is fatal in 10%–20% of patients (*1*,*4*). Misidentification of toxic mushroom species can lead to inadvertent ingestion involving multiple family members when communal meals are prepared.

Characterization of the outbreak was complicated by likely underreporting of milder cases and substantial amounts of missing data regarding the amount of mushroom eaten and the patients’ clinical course. Many patients affected by this outbreak spoke languages other than English and reported that the mushrooms they ate resembled edible varieties in other countries, highlighting the need for multilingual, culturally relevant public health outreach.

Foraging for mushrooms is a major risk factor for poisoning by amatoxin-containing mushrooms (*5*). The species that causes the most deaths,* A. phalloides* (also known as the death cap mushroom), is endemic to Europe and is believed to have entered the United States in contaminated soil (*6*). The similarly toxic *A. ocreata *is native to California and is found throughout the state. Along the U.S. west coast, *A. phalloides* and *A. ocreata* can be found in a range of environments, including urban parks and the undisturbed coastal live oak woodlands. Consequently, persons living or foraging in these areas are at increased risk for poisoning from eating amatoxin-containing mushrooms. Although smaller blooms can occur year-round, the combined peak season for both species is October–April. Given that large blooms were reported within during period in 2025–2026, the increased risk for accidental consumption could persist longer than previously anticipated.

Amatoxins are not denatured by cooking or other methods of food preparation and result in delayed onset of signs and symptoms, which can contribute to fatal outcomes (*1*). Amatoxins are readily absorbed from the gastrointestinal tract and taken up into liver cells, where they impair protein synthesis, leading to liver cell death and fulminant liver failure (*1*,*7*,*8*). Poisoning occurs in three phases (*7*,*8*). The first phase is characterized by delayed onset of abdominal pain, nausea, vomiting, and diarrhea, often occurring >6 hours after ingestion. Patients might have no laboratory evidence of liver injury at this time. Early identification and treatment are critical for improved outcomes; however, *Amanita* toxicity can be missed if a history of mushroom ingestion is not identified. The second phase, occurring 12–36 hours after ingestion, is characterized by laboratory evidence of liver injury, coagulopathy, and acute kidney injury. The third phase occurs 2–6 days after ingestion and is marked by worsening liver function that can progress to fulminant liver and kidney failure. Most patients recover; however, some require liver transplantation. The modern case-fatality rate is 10%–20%, based on prior case series (*9*).

Despite the severity and potential lethality of amatoxin-containing mushroom poisoning, no standardized treatment regimens are available, and no Food and Drug Administration (FDA)-approved therapies exist. Because amatoxin is eliminated by the kidneys, aggressive IV hydration is recommended. Some evidence, although weak, exists to support the use of activated charcoal,^†^ polymyxin B, cyclosporine,^§^ and octreotide^¶^ and the performance of biliary drainage** in amatoxin poisoning (*8*). The three most commonly used antidotes are N-acetylcysteine^††^ and high-dose penicillin G,^§§^ which are both used off-label (i.e., for indications not listed on the official label), and silibinin,^¶¶^ a potent antioxidant and hepatoprotective compound (*8*). CPCS medical directors developed a protocol that provides guidance on initial management, including the acquisition of silibinin (CPCS, unpublished document, 2025). This experimental therapy is not routinely stocked in hospitals but is available through the FDA Emergency Investigational New Drug program in coordination with the manufacturer. Urine amatoxin testing is limited to specialized laboratories; no FDA-approved clinical test to confirm amatoxin ingestion is available, although a commercially available point-of-care test has been developed (*10*). Identification of *Amanita* mushroom exposure requires eliciting the appropriate clinical history (e.g., eating foraged wild mushrooms), mushroom identification by a mycologist, and other field tests (e.g., spore prints***).

The serious illness and potential death associated with amatoxin-containing mushroom ingestions represent serious public health concerns and warrant 1) outreach to communities about the dangers of eating foraged wild mushrooms and 2) education for medical providers to improve recognition of amatoxin mushroom poisoning in patients with nonspecific gastrointestinal symptoms and hepatotoxicity. In addition, poison control centers provide real-time clinical guidance on the management of individual patients and serve as an early warning system for emerging public health threats. Heightened awareness is important during the fall months, when increased rainfall and warmer temperatures lead to larger mushroom blooms and increase the risk for accidental exposure. To support urgent public health actions and increase awareness among clinicians, CDPH is working to designate amatoxin poisoning as a reportable condition in California.

## Footnotes

* 45 C.F.R. part 46.102(l)(2), 21 C.F.R. part 56; 42 U.S.C. Sect. 241(d); 5 U.S.C. Sect. 552a; 44 U.S.C. Sect. 3501 et seq.

† Activated charcoal is a porous adsorbent agent that binds drugs and toxins in the gastrointestinal tract with the goal of reducing their systemic absorption.

§ Cyclosporine is a known, highly potent, inhibitor of the organic anion transporting polypeptide 1B3 (OATP1B3) liver uptake transporter and is hypothesized to decrease the uptake of amatoxin into liver cells.

¶ Octreotide binds somatostatin receptors coupled to phospholipase C through G proteins, leading to smooth muscle contraction in the blood vessels and possible prevention of gallbladder emptying, which might reduce the recirculation of amatoxins in bile to the liver.

** Biliary drainage is a procedure that involves either aspirating bile or placing a drain into the bile ducts with the goal of interrupting enterohepatic recirculation.

†† N-acetylcysteine is a glutathione precursor with demonstrated efficacy as treatment for acetaminophen poisoning. Its use in *Amanita* species mushroom poisoning is thought to be related to its antioxidant and liver-protective properties, although data on efficacy are inconclusive.

§§ Penicillin G is thought to displace amatoxin from albumin, by blocking its uptake from liver cells, binding circulating amatoxins, and preventing α-amanitin binding to RNA polymerase.

¶¶ Silibinin dihemisuccinate, a milk thistle extract, competitively inhibits amatoxin uptake by the liver and enterohepatic recycling. It is available in the United States through an Emergency FDA Investigational New Drug application.

*** Spore prints, used to aid with mushroom identification, are created by placing a freshly harvested mushroom on paper and leaving it for ≤24 hours to obtain a pattern of the spores.
